# Prognostic value of systemic inflammatory response index in patients with bladder cancer: a systematic review and meta-analysis

**DOI:** 10.3389/fonc.2026.1876253

**Published:** 2026-09-14

**Authors:** Dikai Mao, Ruilin Chen, Feifei Guan, Ji Sun, Runmiao Hua, Jiaguo Huang

**Affiliations:** 1 Department of Urology, Affiliated Xiaoshan Hospital, Hangzhou Normal University, Hangzhou, China; 2 The Second School of Clinical Medicine, Zhejiang Chinese Medical University, Hangzhou, China; 3 Physical Examination Center, Affiliated Xiaoshan Hospital, Hangzhou Normal University, Hangzhou, China

**Keywords:** bladder cancer, meta-analysis, prognosis, systematic review, systemic inflammatory response index

## Abstract

**Background:**

The systemic inflammatory response index (SIRI) has been confirmed to be associated with the prognosis of various solid tumors. The predictive value of the preoperative SIRI on the prognosis of bladder cancer (BC) patients remains unknown. This systematic review and meta-analysis investigated the association between preoperative SIRI and the prognosis of BC patients.

**Methods:**

Relevant studies were retrieved from PubMed, Web of Science, Embase, the Cochrane Library, Chinese National Knowledge Infrastructure, Wan Fang database, and VIP Chinese Science and Technology Periodicals Database from inception to April 18, 2026. The prognostic value of preoperative SIRI for BC patients was assessed using pooled hazard ratios (HRs) and 95% confidence intervals (CIs). Subgroup analyses were performed to investigate potential sources of heterogeneity, and sensitivity analyses were conducted to assess the robustness of the findings.

**Results:**

Fourteen studies comprising 4,098 patients were included. An elevated SIRI was significantly associated with poorer overall survival (OS; HR = 2.20, 95% CI = 1.61-3.01), recurrence-free survival (RFS; HR = 2.13, 95% CI = 1.74-2.61), and progression-free survival (PFS; HR = 4.06, 95% CI = 2.67-6.15) in BC patients.

**Conclusion:**

SIRI is a predictor of the survival, recurrence and disease progression in BC patients. Further large-scale prospective studies are required to confirm these findings.

**Systematic review registration:**

https://inplasy.com/inplasy-2026-5-0016/, identifier 202650016.

## Introduction

Bladder cancer (BC) is one of the most commonly diagnosed malignancies globally and represents the predominant neoplasm of urinary system. According to the 2022 Global Cancer Statistics, there were approximately 613,791 new cases of BC and 220,349 deaths (1). BC is classified as non-muscle-invasive bladder cancer (NMIBC) and muscle-invasive bladder cancer (MIBC) based on tumor invasion depth. Nearly 70% of the patients are initially diagnosed with disease confined to the mucosa or submucosa (2, 3). Transurethral resection of the bladder tumor (TURBT), complemented with intravesical immunotherapy or chemotherapy, is the gold standard for the treatment of NMIBC (3). Although NMIBC generally has a good prognosis, with a 5-year survival rate of nearly 90%, recurrence occurs in 30–80% of cases, and up to 45% of cases progress to MIBC within five years (4). In contrast, patients with MIBC, who are treated with radical cystectomy (RC) plus neoadjuvant or adjuvant chemotherapy or, more recently, immunotherapy, face a higher risk of cancer-specific mortality compared to those with NMIBC (5). Therefore, easily accessible and objective markers are required to improve risk stratification and accurately predict the prognosis of BC patients.

Numerous studies have reported an association between certain inflammatory markers and the prognosis of BC patients (6–8). One such easily obtainable clinical biomarker is the systemic inflammatory response index (SIRI), which reflects the systemic inflammatory status. Numerous systematic reviews and meta-analyses have synthesized the findings of various studies, providing significant insights into the association between SIRI and various solid tumors, including glioma (9), breast cancer (10), cervical cancer (11), nasopharyngeal carcinoma (12), esophageal cancer (13), gastric cancer (14), colorectal cancer (15), non-small cell lung cancer (16), hepatocellular carcinoma (17), and pancreatic cancer (18). Consistently, multiple evidence demonstrates that elevated SIRI levels are significantly associated with worse prognosis in patients with these types of cancer.

Moreover, SIRI has been identified as an independent predictor of prognosis in urological malignancies. A meta-analysis by Wangbin Ma et al. reported that elevated SIRI predicted poor survival outcomes in patients with urological malignancies, including renal cancer, upper tract urothelial carcinoma, bladder cancer, and prostate cancer (19). Nevertheless, this study grouped different types of tumors into a single category of urological malignancies for pooled analysis, which introduces significant heterogeneity. Furthermore, only three of the included studies specifically focused on BC. Although previous studies have reported an association between SIRI and survival outcomes in BC patients (20–22), no comprehensive meta-analysis specifically focusing on BC has been conducted to date. Based on these findings, this meta-analysis aimed to systematically evaluate the prognostic value of preoperative SIRI in BC patients.

## Methods

### Protocol registration

This systematic review and meta-analysis was performed following the Preferred Reporting Items for Systematic Reviews and Meta-Analyses guideline, and its protocol was registered online on INPLASY (identifier: 202650016) (23).

### Literature search strategy

Relevant literature search was conducted in PubMed, Web of Science, Embase, the Cochrane Library, Chinese National Knowledge Infrastructure, Wan Fang database, and VIP Chinese Science and Technology Periodicals Database from inception to April 18, 2026, without language limitations. The search terms combined the Medical Subject Heading (MeSH) with other related words. The search query in PubMed was presented in **Table 1**. Similar search terms were adopted for the other databases. Further relevant literature was identified by manually examining the references cited in the included articles.

**Table 1:** Search query used for the PubMed database.

Search line	Search query
#1	("Urinary Bladder Neoplasms"[Mesh]) OR ((((((((((((((((((Neoplasm, Urinary Bladder[Title/Abstract]) OR (Urinary Bladder Neoplasm[Title/Abstract])) OR (Bladder Neoplasms[Title/Abstract])) OR (Bladder Neoplasm[Title/Abstract])) OR (Neoplasm, Bladder[Title/Abstract])) OR (Bladder Tumors[Title/Abstract])) OR (Bladder Tumor[Title/Abstract])) OR (Tumor, Bladder[Title/Abstract])) OR (Tumors, Bladder[Title/Abstract])) OR (Neoplasms, Bladder[Title/Abstract])) OR (Urinary Bladder Cancer[Title/Abstract])) OR (Cancer, Urinary Bladder[Title/Abstract])) OR (Bladder Cancer[Title/Abstract])) OR (Bladder Cancers[Title/Abstract])) OR (Cancer, Bladder[Title/Abstract])) OR (Cancer of Bladder[Title/Abstract])) OR (Cancer of the Bladder[Title/Abstract])) OR (Malignant Tumor of Urinary Bladder[Title/Abstract]))
#2	((systemic inflammation response index[Title/Abstract]) OR (systemic inflammatory response index[Title/Abstract])) OR (SIRI[Title/Abstract])
#3	#1 AND #2

### Study selection and data extraction

Inclusion criteria were as follows (1): the association between SIRI and the prognosis of BC patients was reported, using the formula SIRI = (Neutrophil count × Monocyte count)/Lymphocyte count, and all stages of BC were included; (2) patients were classified based on preoperative SIRI levels; (3) at least one of overall survival (OS), recurrence-free survival (RFS), or progression-free survival (PFS) was reported; (4) hazard ratios (HRs) or odds ratios (ORs) with 95% confidence intervals (CIs) were provided, or sufficient data for their calculation were available. Exclusion criteria were as follows: (1) studies were case reports, conference abstracts, editorials, or reviews; (2) studies had insufficient data for extraction on BC patient treatment or outcomes; (3) studies were animal studies; (4) studies were duplicate publications or shared overlapping samples with another included study; (5) the full text was unavailable.

Two investigators (DM and FG) independently screened titles and abstracts to determine article suitability, then examined full texts. For duplicate publications, the version with the most complete data was retained. Disagreements were resolved by consulting a third investigator (JH). The same two investigators extracted data into a Microsoft Excel spreadsheet (Microsoft Corporation, Redmond, WA), recording: first author, publication year, study region, design, number of study centers, mean/median age, sample size, treatment, follow-up duration, SIRI cut-off and its selection method, survival outcomes, and HRs/ORs with 95% CIs.

### Quality assessment

Two investigators (DM and FG) independently assessed study quality using the Newcastle-Ottawa Scale (NOS). Disagreements were resolved by consulting a third investigator (JH). The NOS scores range from 0 to 9, with higher scores indicating better quality. Studies were categorized as high (7-9), moderate (4-6), or low (0-3) quality.

### Study outcomes and statistical analysis

Meta-analysis was conducted using Stata 16.0 software (StataCorp). Pooled HRs with 95% CIs were calculated to assess the association between preoperative SIRI and survival outcomes. Heterogeneity across the included studies was assessed using the Q test and quantified using the *I*^2^ statistic. Based on *I*^2^ value, heterogeneity was categorized as high (>75%), moderate (25%–75%), or low (<25%) (24). A random-effects model was applied when *I*^2^ > 50% and/or *P* < 0.05; otherwise, a fixed-model was used. Subgroup analyses were performed to identify potential sources of heterogeneity. If grouped data on subgroup categories were not available, they were excluded. Studies with unavailable subgroup data were excluded from the respective analyses. Sensitivity analysis was also conducted to evaluate the influence of individual studies on the pooled results. Publication bias was assessed using Egger’s test. However, the power to test for publication bias is limited in a meta-analysis that includes less than 10 studies (25). *P* < 0.05 was considered statistically significant.

## Results

A total of 83 publications were retrieved by searching PubMed (n = 16), Web of science (n = 5), Cochrane Library (n = 4), EMbase (n = 33), Chinese National Knowledge Infrastructure (n = 12), Wan Fang database (n = 8), and VIP Chinese Science and Technology Periodicals Database (n = 5). After duplicate removal, 64 articles were screened by title and abstract, of which 43 proceeded to full-text review. As a whole, 14 studies published between 2021 and 2025, and involving 4,098 participants were ultimately included in this meta-analysis (20, 22, 26–37). The PRISMA flow chart of the study selection is presented in **Figure 1**. Nine studies were conducted in China, three were conducted in Turkey, and two were conducted in Italy. Of the included studies, thirteen was retrospective and one were prospective. Twelve studies were single-center studies and two was multi-center study. The sample size of the included studies ranged from 40 to 1,387. Nine of the studies included patients treated with TURBT, and the other five included patients who underwent RC. The cut-off values for SIRI ranged from 0.6 to 379.61. Furthermore, the association of preoperative SIRI with OS, RFS, and PFS was evaluated in seven, seven, and five studies, respectively. All studies scored ≥6 on the NOS, indicating a high overall quality (**Table 2**). **Tables 1** and **3** present an overview of the baseline data and quality evaluations of the included studies.

**Figure 1:**
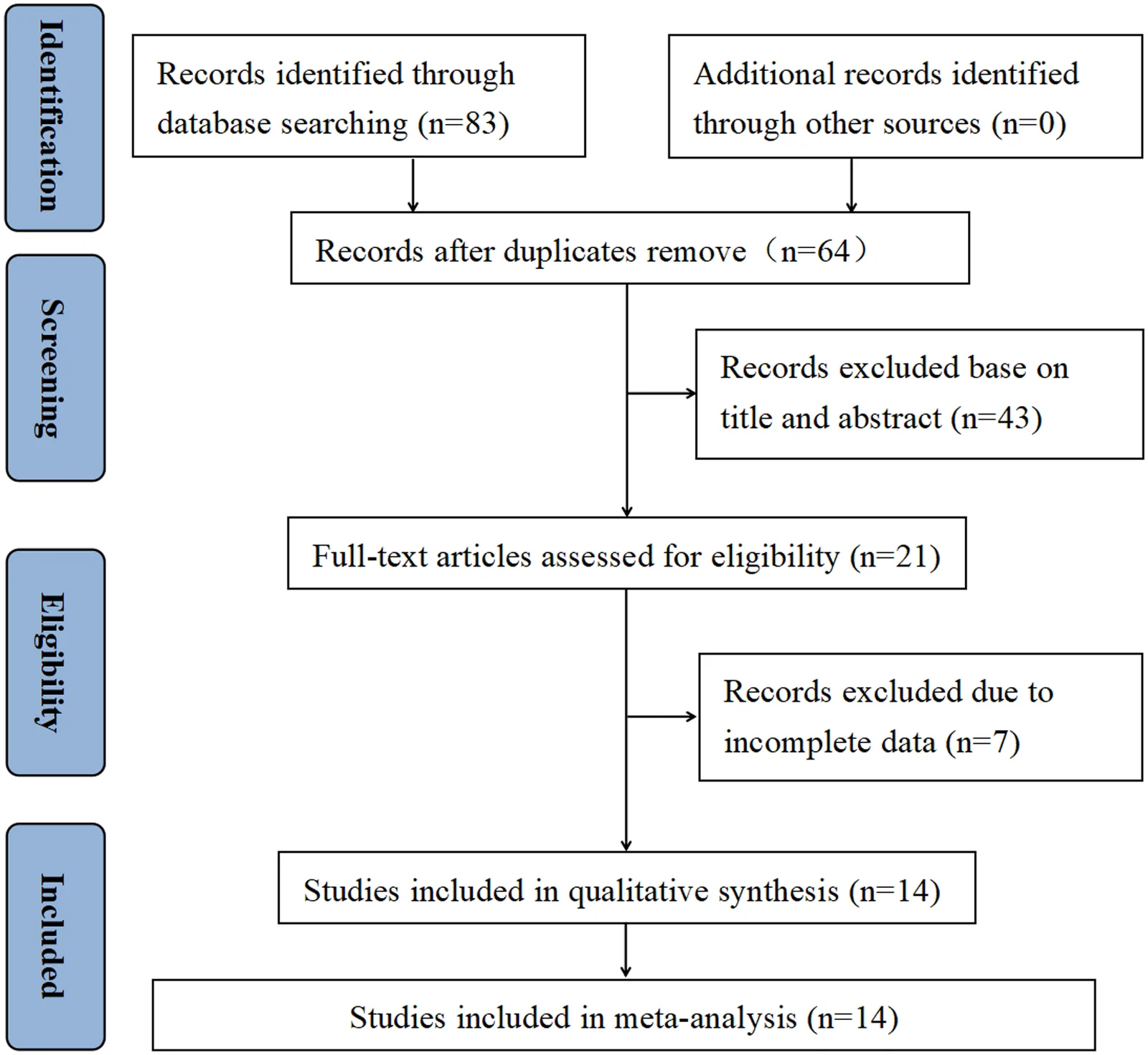
PRISMA diagram showing the study selection process.

**Table 2:** The NOS quality of included studies.

Study	Selection				Comparability	Outcome			Total	Quality
	①	②	③	④	⑤	⑥	⑦	⑧		
Bizzarri, F. P.2025 (20)	1	1	0	1	2	1	1	0	7	high
Eren, A. E.2025 (26)	1	1	0	1	1	1	1	0	6	moderate
Feng, R.2025 (27)	1	1	0	1	2	1	1	0	7	high
Gui, Q.2025 (28)	1	1	0	1	2	1	1	0	7	high
Jianming, P.2023 (29)	1	1	0	1	2	1	1	1	8	high
Jun, D.2023 (30)	1	1	0	1	1	1	1	0	6	moderate
Kayar, R.2025 (31)	1	1	0	1	1	1	1	0	6	moderate
Ming, Y.2023 (32)	1	1	0	1	2	1	1	0	7	high
Nengyong, L.2024 (33)	1	1	0	1	2	1	1	0	7	high
Russo, P.2025 (22)	1	1	0	1	2	1	1	0	7	high
Tian, X.2021 (34)	1	1	0	1	2	1	1	0	7	high
Ye, K.2023 (35)	1	1	0	1	2	1	1	1	8	high
Yilmaz, H.2023 (36)	1	1	0	1	2	1	1	0	7	high
Zhiqiang, Y.2024 (37)	1	1	0	1	2	1	1	1	8	high

NOS, Newcastle-Ottawa Scale; ① Is the case definition adequate; ② Representativeness of the cases; ③ Selection of controls; ④ Definition of controls; ⑤ Comparability of cases and controls on the basis of the design or analysis (a maximum of two sores can be given for Comparability); ⑥ Ascertainment of exposure; ⑦ Same method of ascertainment for cases and controls; ⑧ Nonresponse rate.

**Table 3:** An overview of the baseline data of the included studies.

Study	Region	Study design	Number of study centers	Treatment	Sample size	Age (years)	Follow-up (months)	SIRI cut-off	cut-off selection	Survival outcome	HR	LCI	UCI
Bizzarri, F. P.2025 (20)	Italy	Retrospective	Single-center	TURBT	285	73.2 (34–97)	72	1.12	ROC	RFS	2	1.25	2.5
Eren, A. E.2025 (26)	Turkey	Retrospective	Single-center	TURBT	119	72 (46–96)	–	1.26	ROC	OS	3.419	1.302	8.978
										PFS	3.238	1.236	8.482
Feng, R.2025 (27)	China	Retrospective	Multicenter	RC	1387	–	31(IQR: 12.8 – 62.1)	2.53	ROC	OS	2.81	2.32	3.41
Gui, Q.2025 (28)	China	Retrospective	Single-center	TURBT	158	–	As of October, 2024	0.716	ROC	PFS	2.979	1.11	8.027
Jianming, P.2023 (29)	China	Prospective	Single-center	TURBT	101	55.73 ± 6.68	12	379.61	ROC	RFS	3.714	1.269	10.864
Jun, D.2023 (30)	China	Retrospective	Single-center	TURBT	103	59.71 ± 11.34	As of May, 2023	0.725	ROC	PFS	3.102	1.149	8.371
Kayar, R.2025 (31)	Turkey	Retrospective	Multicenter	RC	115	65 (60–71)	Ranged from 24 to 123	1.5	ROC	OS	3.074	2.19	4.32
Ming, Y.2023 (32)	China	Retrospective	Single-center	TURBT	235	–	60	0.78	Median	RFS	1.824	1.048	3.174
Nengyong, L.2024 (33)	China	Retrospective	Single-center	TURBT	40	65	16	1.46	ROC	RFS	12.6	1.314	120.832
Russo, P.2025 (22)	Italy	Retrospective	Single-center	RC	228	79 (73–83)	26 months(IQR: 10–54)	1.71	ROC	OS	1.34	0.9	2.02
										RFS	1.62	1.03	2.52
Tian, X.2021 (34)	China	Retrospective	Single-center	TURBT	112	–	As of June, 2020	1.18	Median	OS	6.397	1.298	31.529
										PFS	3.696	1.441	10.932
Ye, K.2023 (35)	China	Retrospective	Single-center	TURBT	540	63 (53–70)	As of December, 2020	0.785	ROC	RFS	4.43	2.632	7.455
										PFS	7.034	3.245	15.244
Yilmaz, H.2023 (36)	Turkey	Retrospective	Single-center	RC	241	65 (60–70)	20months(IQR: 9–52)	1.91	X-tile software	OS	1.94	1.32	2.85
										RFS	2.43	1.46	4.02
Zhiqiang, Y.2024 (37)	China	Retrospective	Single-center	RC	434	–	As of January 30, 2021	0.6	ROC	OS	1.3	1.1	3.1

HR, hazard ratio; IQR, interquartile range; LCI, lower confidence interval; NOS, Newcastle-Ottawa Scale; OS, overall survival; PFS, progression-free survival; RC, radical cystectomy; RFS, recurrence-free survival; ROC, receiver operating characteristic curve; SIRI, systemic inflammatory response index; TURBT, transurethral resection of the bladder tumor; UCI, upper confidence interval.

### Association of preoperative SIRI and OS in BC patients

Seven studies reported the association between preoperative SIRI and OS in BC patients (22, 26, 27, 31, 34, 36, 37). A random effects model (*I*^2^ = 71.6%, *P* = 0.002) was conducted, and the pooled analysis showed that elevated SIRI was significantly associated with poorer OS (HR = 2.20, 95% CI = 1.61–3.01) (**Figure 2**). Sensitivity analysis showed that removal of any individual study did not materially alter the results, indicating that the findings from the random-effects model were stable and reliable (**Figure 3**). Given the substantial heterogeneity observed in the overall meta-analysis (*I*^2^ = 71.6%), we further explored the potential sources of heterogeneity based on region, number of study centers, simple size, surgical treatment, cut-off selection, and study quality. **Table 4** indicated that number of study centers (single-center vs. multi-center) was the primary source of heterogeneity (P < 0.001 between subgroups). Finally, Egger’s tests were conducted to evaluate publication bias, showing no statistically significant publication bias (*P* = 0.672).

**Figure 2:**
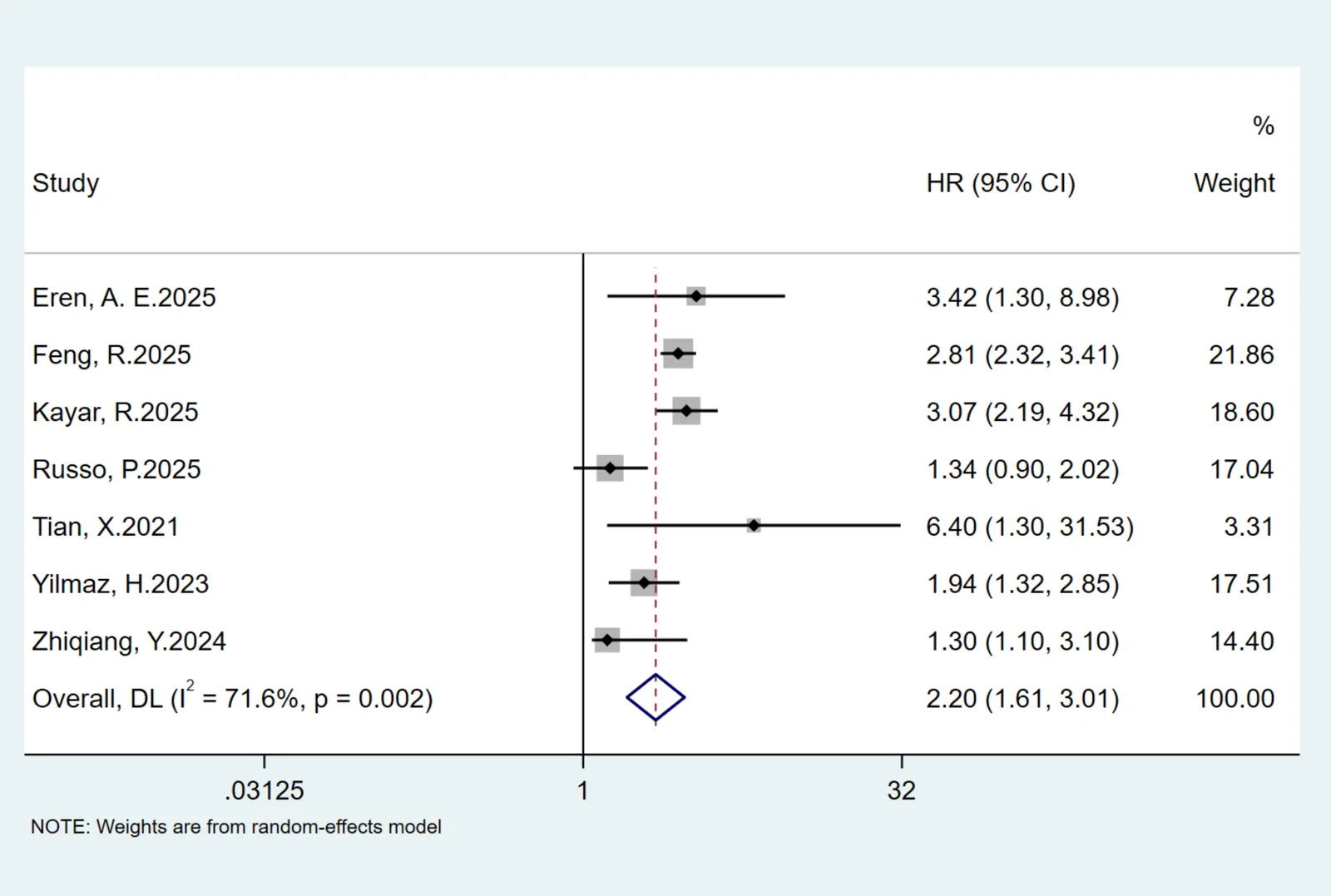
Forest plots of the association of SIRI and OS in BC patients.

**Figure 3:**
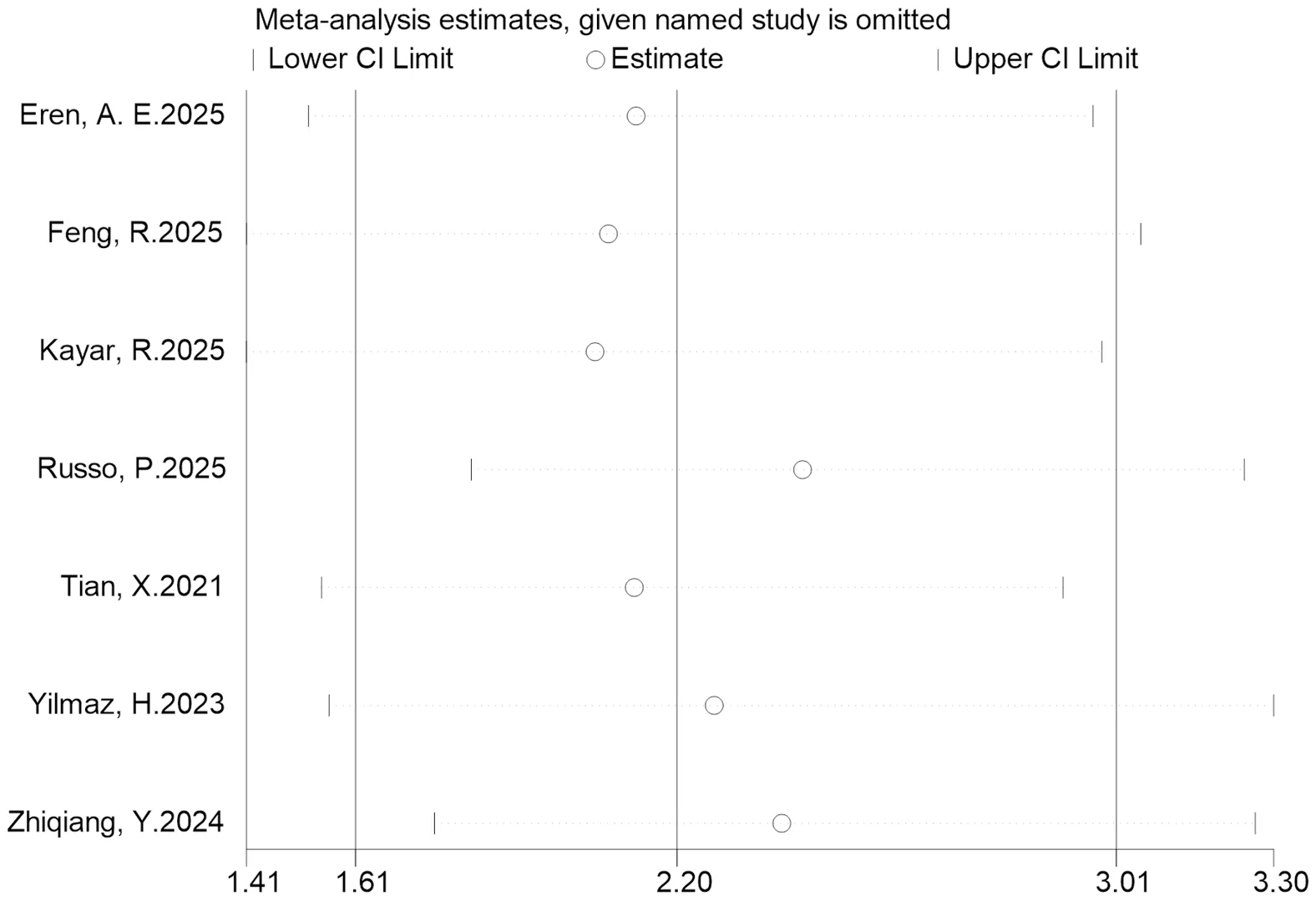
Sensitivity analyses for the association of SIRI and OS in BC patients.

**Table 4:** Subgroup analyses for the prognostic role of SIRI for OS in BC patients.

Subgroups	Items	Number of studies	HR (95% CI)	*I* ^2^	*P* for heterogeneity	Heterogeneity between groups
Country	Europe	4	2.16 (1.41-3.32)	71.9%	0.014	0.859
	Asia	3	2.33 (1.18-4.60)	77.1%	0.178	
Number of study centers	Single-center	5	1.67 (1.32-2.12)	46.4%	0.114	<0.001
	Multicenter	2	2.87 (2.43-3.40)	0	0.652	
Simple size	≤228	4	2.57 (1.39-4.76)	74.8%	0.008	0.534
	>228	3	2.02 (1.31-3.12)	78.0%	0.011	
Surgical treatment	RC	5	2.04 (1.46-2.86)	79.1%	<0.001	0.132
	TURBT	2	4.04 (1.77-9.24)	0	0.510	
Cut-off selection	ROC	5	2.16 (1.48-3.16)	78.3%	0.001	0.690
	Non-ROC	2	2.71 (0.95-7.76)	50.8%	0.154	
Study quality	High	5	1.94 (1.29-2.92)	77.7%	0.001	0.075
	Moderate	2	3.11 (2.26-4.28)	0	0.839	

CI, confidence interval; HR, hazard ratio; OS, overall survival; RC, radical cystectomy; ROC, receiver operating characteristic curve; SIRI, systemic inflammatory response index; TURBT, transurethral resection of the bladder tumor.

### Association of preoperative SIRI and RFS in BC patients

Seven studies reported the association between preoperative SIRI and RFS in BC patients (20, 22, 29, 32, 33, 35, 36); the pooled analysis showed that elevated SIRI was associated with poorer RFS (HR = 2.13, 95% CI = 1.74–2.61), based on a fixed effects model (*I*^2^ = 15.1%, *P* = 0.314) (**Figure 4**). Sensitivity analysis indicated that sequential removal of individual studies did not alter the overall results, confirming the stability and reliability of the fixed-effects model findings (**Figure 5**). Given the low heterogeneity across the included studies, no subgroup analysis or meta-regression was performed. Egger’s tests suggested the presence of publication bias (*P* = 0.023).

**Figure 4:**
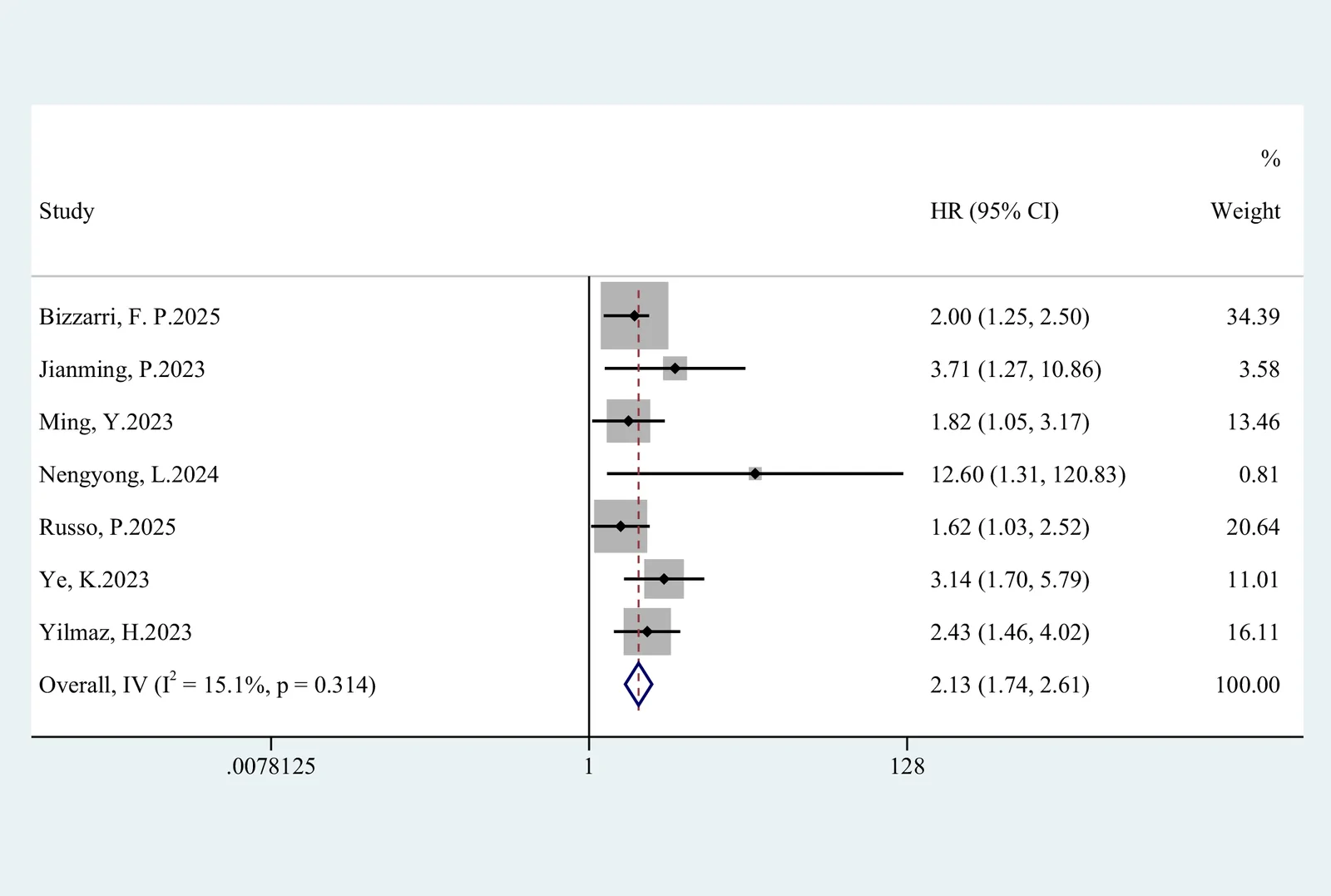
Forest plots of the association of SIRI and RFS in BC patients.

**Figure 5:**
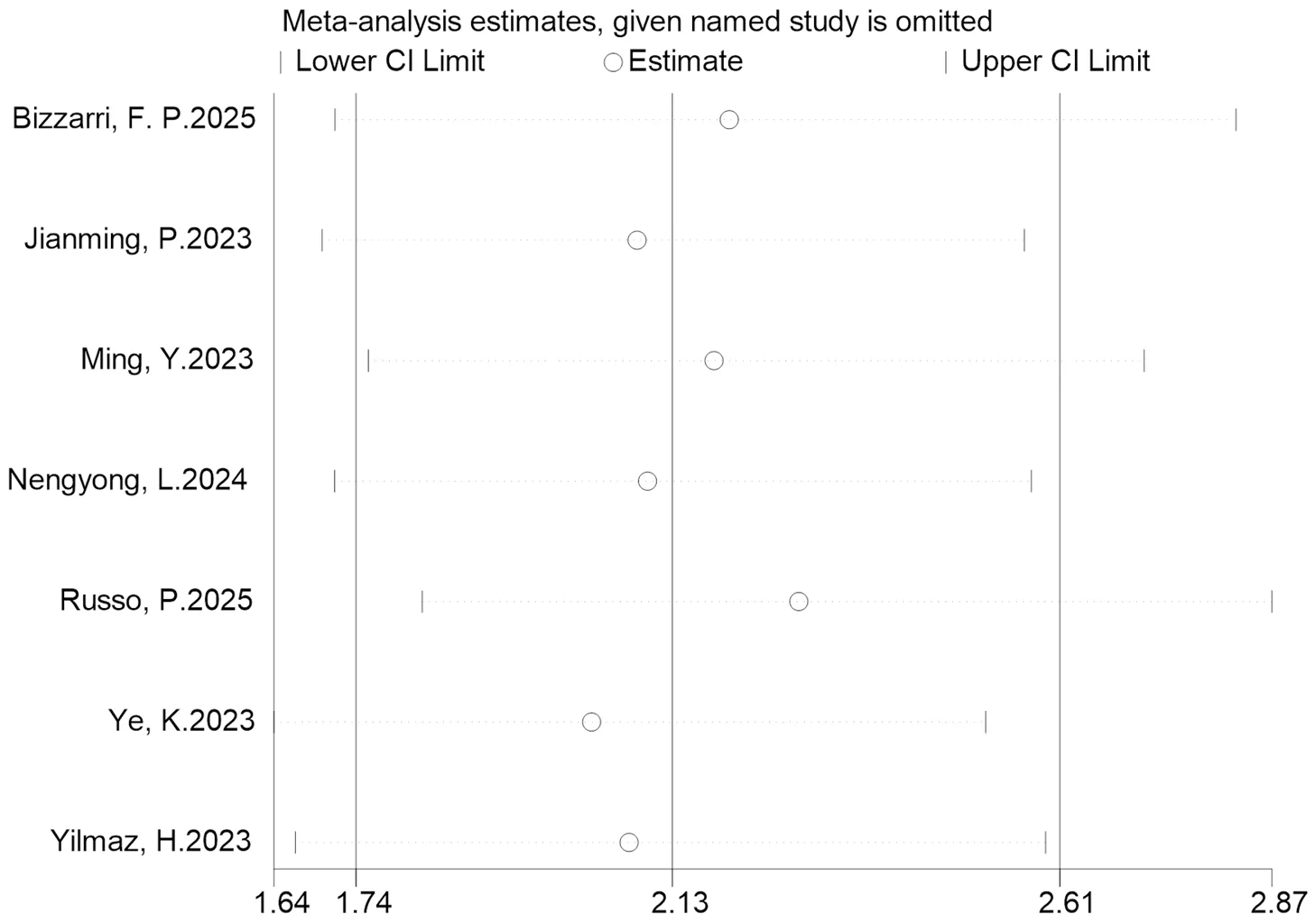
Sensitivity analyses for the association of SIRI and RFS in BC patients.

### Association of preoperative SIRI and PFS in BC patients

Five studies reported the association between preoperative SIRI and PFS in BC patients (26, 28, 30, 34, 35); the pooled analysis showed that elevated SIRI was associated with poorer PFS (HR = 4.06, 95% CI = 2.67–6.15), based on a fixed effects model (*I*^2^ = 0%, *P* = 0.585) (**Figure 6**). Sensitivity analysis revealed that the arbitrary deletion of studies did not affect the results, indicating that the results are stable and reliable, as shown in **Figure 7**. Due to the limited number of included studies and the low heterogeneity observed, no subgroup analysis or meta-regression was performed, nor was publication bias assessed.

**Figure 6:**
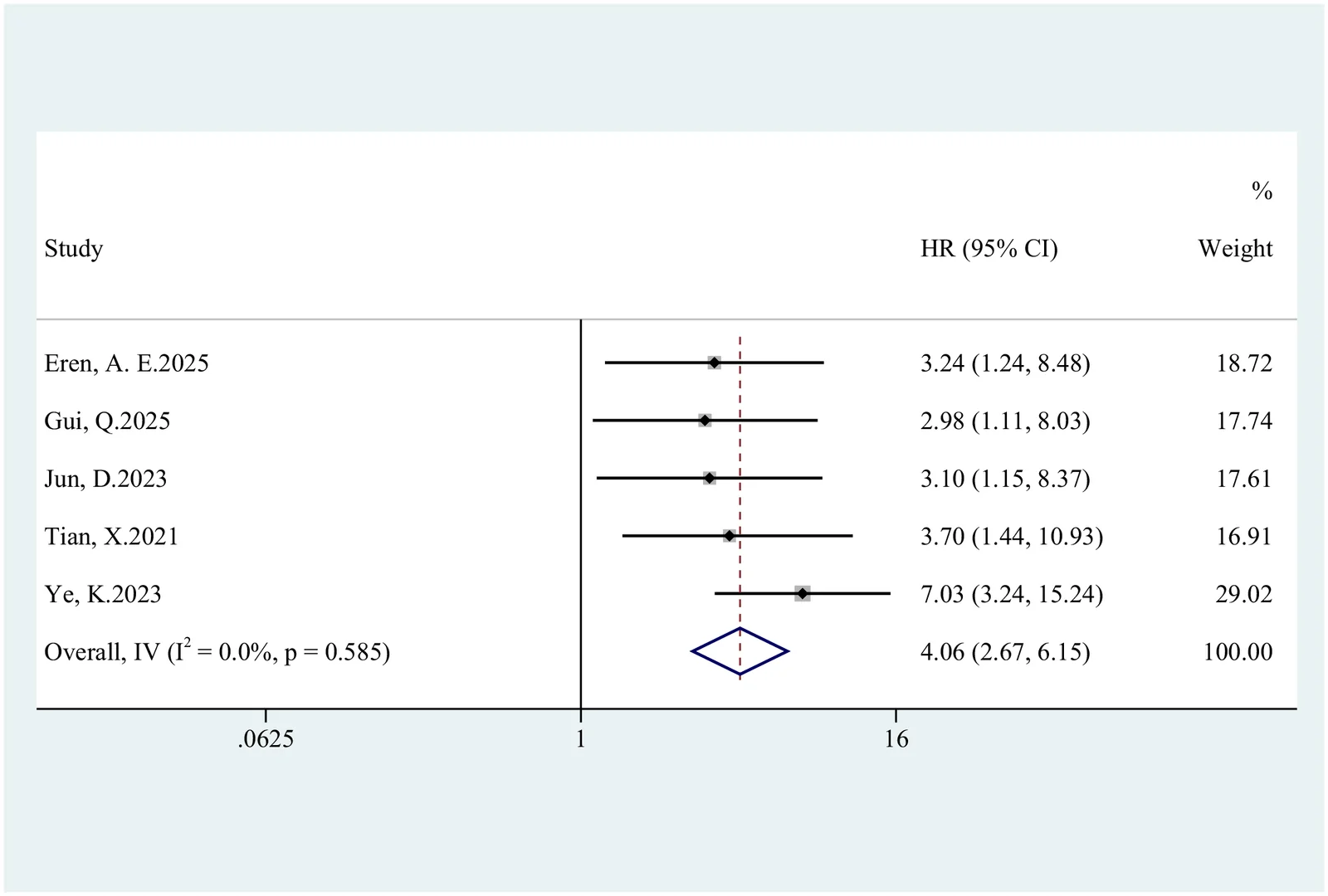
Forest plots of the association of SIRI and PFS in BC patients.

**Figure 7:**
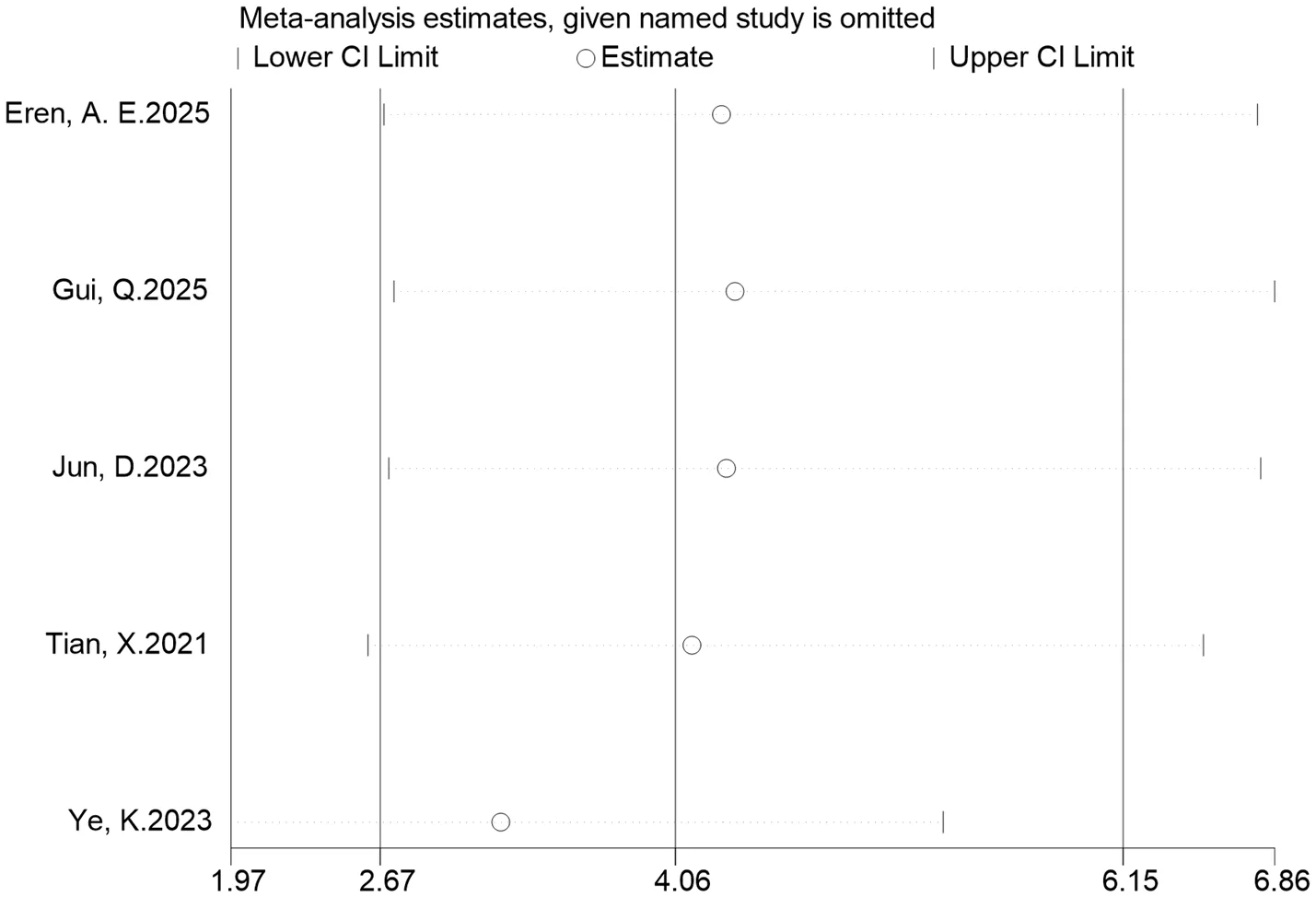
Sensitivity analyses for the association of SIRI and PFS in BC patients.

## Discussion

To our knowledge, this is the first meta-analysis to comprehensively evaluate the association between preoperative SIRI and clinical outcomes in BC patients. Our pooled analysis demonstrated that elevated SIRI was significantly associated with poorer OS (HR = 2.20), RFS (HR = 2.13), and PFS (HR = 4.06). These findings position preoperative SIRI as a robust prognostic biomarker. Notably, because SIRI is derived from routine complete blood counts, its clinical application offers a cost-effective strategy for risk stratification and follow-up guidance in BC patients without incurring additional healthcare costs. The SIRI was calculated using the following formula: SIRI = (Neutrophil count × Monocyte count)/Lymphocyte count. Consequently, elevated SIRI might result from increased neutrophils, increased monocytes, and/or decreased lymphocytes. Several potential mechanisms may explain its predictive role in cancer.

The recruitment and activation of stromal cells, adipocytes, fibroblasts, inflammatory cells, and mesenchymal progenitors can alter the tumor microenvironment, thereby promoting the development, invasion, proliferation, and metastasis of BC (38, 39). The predictive value of SIRI in carcinogenesis and tumor progression may be linked to the functions of neutrophils, a key component of the SIRI formula. Upon an increase in neutrophil count, these cells interact with other populations to produce cytokines and effector molecules (40). Within the tumor microenvironment, neutrophils can polarize into either antitumorigenic or protumorigenic phenotypes, thereby modulating immune responses (41, 42). They are rapidly recruited to sites of inflammation by chemotactic signals, including chemokines and lipid mediators, to mediate host defense (43). Their major pro-tumor activities encompass promoting cancer cell proliferation, invasion, metastasis, extracellular matrix remodeling, angiogenesis, lymphangiogenesis, and suppressing antitumor immune surveillance (44). Collectively, these neutrophil-driven processes contribute to tumor formation and progression (45, 46). Ly6C (high) monocytes serve as the primary circulating precursors that continuously infiltrate tumors and give rise to all non-proliferating tumor-associated macrophage (TAM) subsets (47). Monocytes are recruited from the bloodstream into tumors, where they differentiate into diverse subpopulations of TAMs that promote tumor progression, angiogenesis, invasion, metastasis, and immunosuppression (48).

Besides, lymphocytes participate in immune defense by inducing the death of cytotoxic cells and inhibiting the proliferation and migration of tumor cells (49, 50). Lymphocyte infiltration in tumor tissues is associated with improved therapeutic outcomes. Conversely, a decrease in lymphocyte numbers within the tumor microenvironment impairs anti-tumor immunity, leading to immunosuppression and tumor escape (51). Hence, elevated SIRI driving by increased neutrophil and monocyte counts and decreased lymphocyte count is associated with poor prognosis in cancer patients. Consequently, patients with elevated SIRI levels may require more aggressive therapy and closer follow-up for timely detection of tumor recurrence and progression.

In the process of literature search, we also identified three studies that reported the the association between preoperative SIRI and major complications after RC in BC patients (21, 52, 53). All three studies employed the Clavien-Dindo Classification (CDC) for standardized reporting of postoperative complications. In the studies by Cinar et al. and Dama et al., SIRI was identified as an independent predictor of severe complications, with endpoints set at 90 days (CDC ≥ IIIb) and 30 days (CDC ≥ IIIa), respectively (OR = 1.28, *P* = 0.03; OR = 1.37, *P* = 0.045) (21, 52). In contrast, the study by Veerakulwatana et al., which evaluated 30-day major complications (CDC ≥ 3–5), did not demonstrate a significant predictive role for SIRI (OR = 1.01, *P* = 0.986) (53). Notable heterogeneity exists across these studies, particularly with regard to the observation time window for complications (30 days vs. 90 days) and the severity thresholds (CDC ≥ IIIa vs. ≥ IIIb vs. 3–5). Consequently, given the substantial heterogeneity of the current evidence and the limited number of available studies, performing a conventional meta-analysis remains challenging. Evidence on SIRI for predicting RC complications is insufficient and conflicting. Future research should adopt standardized designs with consistent endpoints (e.g., 90 days CDC ≥ IIIa) to clarify SIRI’s clinical value in RC perioperative risk stratification.

However, there are several limitations in this meta-analysis that need to be recognized. First, because most of the included studies were retrospective, the presence of unmeasured or uncontrolled confounding factors may have influenced the accuracy and reliability of our findings. Second, the included studies were predominantly derived from four countries, namely China, Turkey, and Italy, which introduces geographical constraints and may limit the generalizability of our conclusions to other populations or regions. Third, although all participants were patients with BC, there was considerable heterogeneity across studies regarding baseline characteristics, clinical stage, histological subtype, tumor invasion degree, and treatment regimens. For instance, the prognosis varies markedly among different histological subtypes, and previous literature has shown that high-grade lesions have a significantly higher risk of recurrence and progression than low-grade lesions (54, 55). Due to the relatively small sample size, we were unable to account for all confounding factors through subgroup analysis. It should be noted that all stages of BC (including both NMIBC and MIBC) were incorporated in the pooled analysis. Owing to the unavailability of stage-stratified data in the included studies, we were unable to perform subgroup analyses based on tumor stage. Instead, we used surgical treatment as a grouping variable in the subgroup analysis to partially mitigate this limitation, given that surgical treatment is closely correlated with clinical staging. Elevated SIRI was identified as a risk factor for BC patients in both the TURBT and RC groups, with no statistically significant difference between the groups. However, this remains an indirect adjustment, and the lack of direct stage-specific stratification constitutes a notable limitation of the present study. Accordingly, further validation in stage-defined cohorts is warranted. Fourth, the criteria for selecting cutoff values differed across studies. Therefore, standardized methods or cutoff values should be thoughtfully adapted to suit different populations or clinical settings. Fifth, publication bias should also be considered. Finally, significant heterogeneity was observed in the analyses of OS; however, subgroup analyses were performed and identified potential sources of this heterogeneity. Prospective studies with larger sample sizes are required to validate our findings and provide more reliable evidence.

## Conclusion

This meta-analysis demonstrated that SIRI is a predictor of the survival, recurrence and disease progression in BC patients. SIRI, being cost-effective and practical, can be utilized to guide the management and follow-up of BC patients. However, further large-scale prospective studies are required to confirm these findings.

## Data Availability

The original contributions presented in the study are included in the article/supplementary material. Further inquiries can be directed to the corresponding authors.
